# Dataset on the global patent networks within and between vehicle powertrain technologies — Cases of ICEV, HEV, and BEV

**DOI:** 10.1016/j.dib.2019.105017

**Published:** 2019-12-19

**Authors:** Amir Mirzadeh Phirouzabad, David Savage, James Juniper, Karen Blackmore

**Affiliations:** a Faculty of Law & Business, The University of Newcastle, 409 Hunter Street, Newcastle, NSW, 2300, Australia; b School of Electrical Engineering and Computing, The University of Newcastle, University Drive, Callaghan, NSW, 2308, Australia

**Keywords:** Battery electric vehicle, Hybrid electric vehicle, Internal combustion engine vehicle, Patents network, Inter-organisational collaboration, International patent classification, Knowledge domain, Environmental innovation

## Abstract

The emergence of networks is a crucial channel for automotive organisations to build and diffuse the required environmental innovations in the transportation sector and accelerate the transition to the green mobility economy. This article contains the dataset regarding the global patents networks shaped both within and between the three vehicle powertrains of internal combustion engine vehicle (ICEV), hybrid electric vehicle (HEV) and battery electric vehicle (BEV) for the period of 1985–2016. The data was acquired from Thomson Reuters' Derwent Innovations Index (DII) platform using the elements of ‘patent families’ and ‘priority dates’. We describe the dataset for the three major automotive periods of ‘towards sustainable mobility’ (1985–1996), ‘towards hybridisation’ (1997–2007), and ‘towards mass commercialisation’ (2008–2016). The dataset bears on two levels, individual and mutual, and we used a separate combined search strategy of keywords and IPCs codes (international patent classification) for each level. At individual level, we explored the internal network features of each powertrain individually (i.e. ICEV, HEV, and BEV). Monitoring a total of 78,732 patents in the three individual powertrain networks, we discovered a total of 1856 unique parent organisations connecting vis-à-vis 5849 bilateral relationships and operating around 4450 joint patents. At mutual level, we explored the mutually common network features of the powertrains (i.e. ICEV-HEV, HEV-BEV, and BEV-ICEV). Monitoring a total of 4702 patents in the three mutual powertrain networks, we discovered a total of 102 unique parent organisations connecting vis-à-vis 384 bilateral relationships and operating around 303 joint patents. These organisations were found specialised around 435 unique subgroup-level IPC codes, of which 134 codes were related to environmentally friendly innovations. The dataset presented in this article is used in [1] and allows researchers not only to map and model the network dynamics and structures within and between the powertrains at global level, but also to analyse and forecast their knowledge flows, technical domains and environmental innovations aspect, using a wide range of models such as social network analysis or regression.

**Table:** Specifications Table

Subject	Transportation, Management of Technology and Innovation
Specific subject area	Vehicle powertrains, Electric vehicles, Networks, Collaborations, Patent bibliometrics
Type of data	CSV files, and figures and tables in the article.
How data were acquired	Data were acquired from Thomson Reuters' Derwent Innovations Index.
Data format	Raw and processed data.
Parameters for data collection	Data collection occurred in November of 2018. We collected data regarding the global patents networks shaped both within and between ICEV, HEV, and BEV for the three periods of 1985–1996, 1997–2007, and 2008–2016. Using separate combined search strategies of keywords and IPCs, the data were extracted and processed based on ‘patent families’ and ‘priority date’.
Description of data collection	Dataset bears on two levels. At individual level (e.g. BEV, or HEV), three individual networks were found with a total of 1856 unique parent organisations connecting vis-à-vis 5849 bilateral relationships while operating around 4450 joint patents. At mutual level (e.g. HEV-BEV), three networks were found with a total of 102 unique parent organisations connecting vis-à-vis 384 bilateral relationships while operating around 303 joint patents. The organisations were found specialised around 435 subgroup-level IPC codes, of which 134 codes were related to environmentally friendly innovations.
Data source location	The University of Newcastle, 409 Hunter Street, Newcastle, NSW, 2300, Australia
Data accessibility	With the article
Related research article	Mirzadeh Phirouzabadi, A., Juniper, J., Savage, D., Blackmore, K., Supportive or inhibitive? —Analysis of dynamic interactions between the interorganisational collaborations of vehicle powertrains, Journal of Cleaner Production, in press, https://doi.org/10.1016/j.jclepro.2019.118790

**Table:** 

**Value of the Data** • A comprehensive database is provided regarding the patents networks that have been shaped both within and between the three powertrains of ICEV, HEV, and BEV at global level for the three major automotive periods of 1985–1996, 1997–2007, and 2008–2016. • This dataset is valuable for researchers interested not only in mapping and modelling the network dynamics and structures within and between the vehicle powertrains on a global scale, but also in analysing and forecasting the complexity and advancement of their knowledge domains and environmental innovations. • The dataset can be employed and analysed by a wide range of models such as social network analysis and regression models. • The dataset can be completed or extended either by collecting collaboration data other than joint patents such as joint ventures and alliances, or by including other powertrain alternatives such as fuel cell vehicles (FCV).

## 1 Data

This article contains and describes a dataset at global scale regarding the patents networks that have been shaped both within and between the three vehicle powertrains of internal combustion engine vehicle (ICEV), hybrid electric vehicle (HEV) and battery electric vehicle (BEV). While the dataset timeframe is between 1985 and 2016, the data will be described for the three individual periods of 1985–1996, 1997–2007, and 2008–2016.

Our dataset is used in Ref. [1] and bears on two levels, individual and mutual. At individual level, we collected and processed the patent network data within individual powertrain systems. At mutual level, we collected and processed the common patent network data between the powertrain systems. While the tables and figures shown in the following sub-sections outline the various features of our dataset at both individual and mutual levels, the full dataset is attached as Supplementary Appendix.

### 1.1 Data at individual level

At individual level, we extracted a total of 78,732 patents related to individual powertrain systems (i.e. HEV, BEV, and ICEV) in order to explore their internal network features. We discovered a total of 1856 unique parent organisations connecting vis-à-vis 5849 bilateral relationships and operating around 4450 joint patents.

Table 1 shows the absolute and relative number of joint patents within each powertrain system over the entire period. An Excel file is included in the Supplementary appendix of this article, which contains the absolute and relative number of joint patents at individual level between 1985 and 2016.

**Table 1:** The absolute and relative number of joint patents at individual level (1985–2016).

Year	BEV	HEV	ICEV	BEV%	HEV%	ICEV%
1985	0	0	19	0.00%	0.00%	100.00%
1986	0	0	13	0.00%	0.00%	100.00%
1987	0	0	19	0.00%	0.00%	100.00%
1988	1	0	13	7.14%	0.00%	92.86%
1989	1	0	17	5.56%	0.00%	94.44%
1990	1	0	18	5.26%	0.00%	94.74%
1991	0	0	31	0.00%	0.00%	100.00%
1992	3	0	19	13.64%	0.00%	86.36%
1993	8	0	28	22.22%	0.00%	77.78%
1994	6	0	26	18.75%	0.00%	81.25%
1995	4	2	45	7.84%	3.92%	88.24%
1996	5	0	36	12.20%	0.00%	87.80%
1997	13	4	48	20.00%	6.15%	73.85%
1998	12	4	36	23.08%	7.69%	69.23%
1999	31	4	47	37.80%	4.88%	57.32%
2000	42	13	59	36.84%	11.40%	51.75%
2001	25	6	73	24.04%	5.77%	70.19%
2002	20	17	90	15.75%	13.39%	70.87%
2003	32	30	146	15.38%	14.42%	70.19%
2004	37	43	127	17.87%	20.77%	61.35%
2005	34	36	142	16.04%	16.98%	66.98%
2006	32	39	165	13.56%	16.53%	69.92%
2007	54	75	165	18.37%	25.51%	56.12%
2008	59	69	135	22.43%	26.24%	51.33%
2009	107	41	106	42.13%	16.14%	41.73%
2010	166	63	152	43.57%	16.54%	39.90%
2011	246	65	125	56.42%	14.91%	28.67%
2012	225	57	127	55.01%	13.94%	31.05%
2013	154	34	101	53.29%	11.76%	34.95%
2014	108	32	70	51.43%	15.24%	33.33%
2015	44	30	52	34.92%	23.81%	41.27%
2016	27	19	20	40.91%	28.79%	30.30%

Sum	1497	683	2270	33.64%	15.35%	51.01%

Table 2 shows the absolute and relative number of the bilateral relationships shaped among the parent organisations of each powertrain system over the entire period. An Excel file is included in the Supplementary appendix of this article, which contains the absolute and relative number of bilateral relationships at individual level between 1985 and 2016.

**Table 2:** The absolute and relative number of bilateral relationships at individual level (1985–2016).

Year	BEV	HEV	ICEV	BEV%	HEV%	ICEV%
1985	0	0	23	0.00%	0.00%	100.00%
1986	0	0	17	0.00%	0.00%	100.00%
1987	0	0	19	0.00%	0.00%	100.00%
1988	1	0	17	5.56%	0.00%	94.44%
1989	1	0	21	4.55%	0.00%	95.45%
1990	1	0	22	4.35%	0.00%	95.65%
1991	0	0	33	0.00%	0.00%	100.00%
1992	3	0	19	13.64%	0.00%	86.36%
1993	8	0	56	12.50%	0.00%	87.50%
1994	4	0	39	9.30%	0.00%	90.70%
1995	1	2	93	1.04%	2.08%	96.88%
1996	5	0	42	10.64%	0.00%	89.36%
1997	13	4	63	16.25%	5.00%	78.75%
1998	12	4	84	12.00%	4.00%	84.00%
1999	40	4	61	38.10%	3.81%	58.10%
2000	50	13	70	37.59%	9.77%	52.63%
2001	27	20	331	7.14%	5.29%	87.57%
2002	22	17	375	5.31%	4.11%	90.58%
2003	34	30	291	9.58%	8.45%	81.97%
2004	39	47	187	14.29%	17.22%	68.50%
2005	36	40	156	15.52%	17.24%	67.24%
2006	36	41	186	13.69%	15.59%	70.72%
2007	60	83	194	17.80%	24.63%	57.57%
2008	63	94	152	20.39%	30.42%	49.19%
2009	112	41	122	40.73%	14.91%	44.36%
2010	178	73	185	40.83%	16.74%	42.43%
2011	258	67	141	55.36%	14.38%	30.26%
2012	267	61	138	57.30%	13.09%	29.61%
2013	176	34	115	54.15%	10.46%	35.38%
2014	129	32	93	50.79%	12.60%	36.61%
2015	52	35	62	34.90%	23.49%	41.61%
2016	29	23	20	40.28%	31.94%	27.78%

Sum	1657	765	3427	28.33%	13.08%	58.59%

**Fig. 1:**
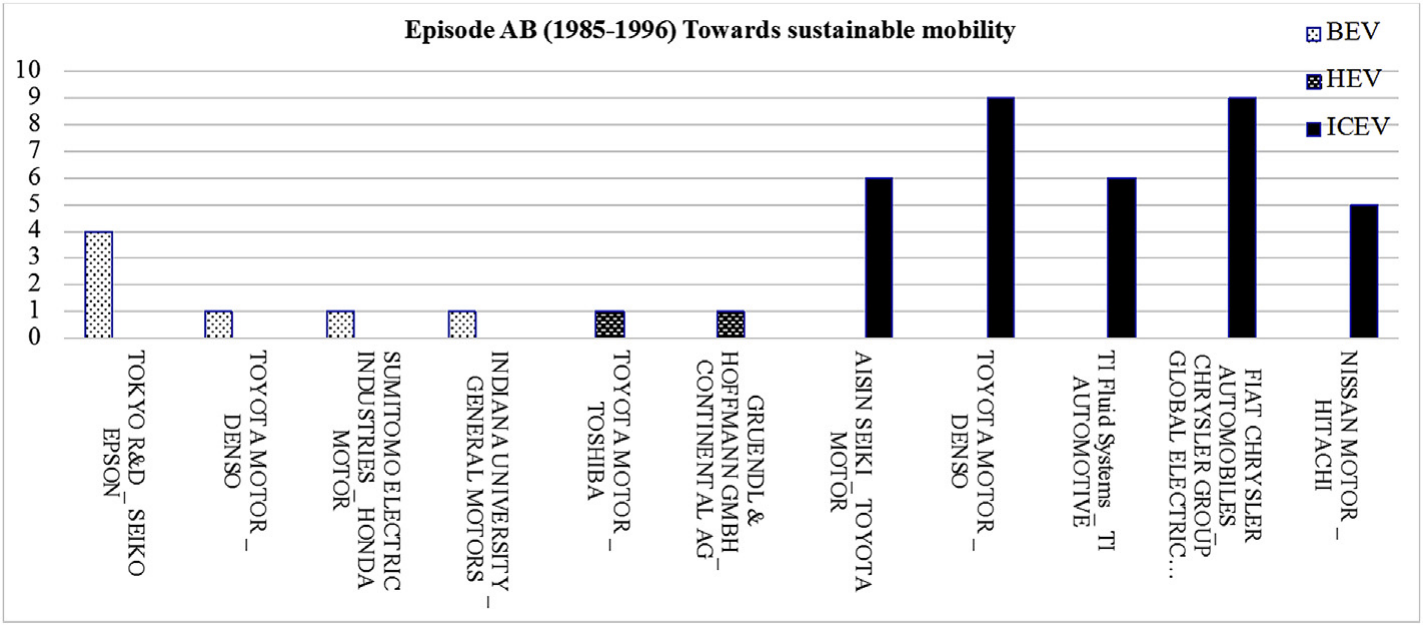
The most frequent bilateral relationships at individual level for the period 1985–1996.

Fig. 1 displays the most frequent bilateral relationships shaped among the parent organisations of each powertrain system for the period 1985–1996. The related raw data lists all the organisations that were in collaboration in the field of individual powertrain systems for the development of joint patents granted between 1985 and 1996.

**Fig. 2:**
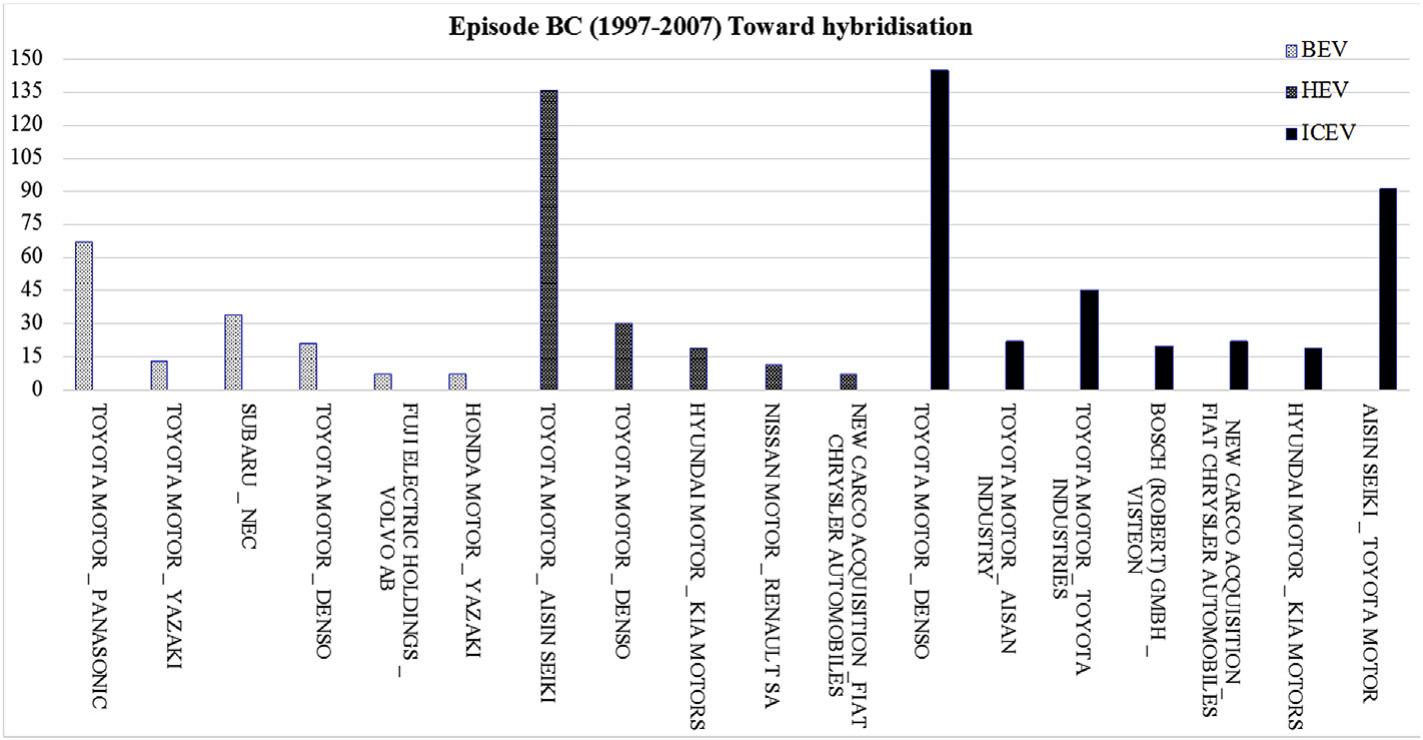
The most frequent bilateral relationships at individual level for the period 1997–2007.

Fig. 2 displays the most frequent bilateral relationships shaped among the parent organisations of each powertrain system for the period 1997–2007. The related raw data lists all the collaborating organisations which developed the joint patents granted between 1997 and 2007 in the field of individual powertrain systems.

**Fig. 3:**
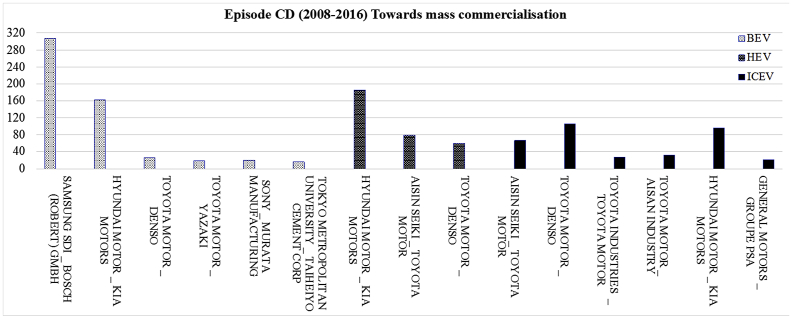
The most frequent bilateral relationships at individual level for the period 2008–2016.

Fig. 3 displays the most frequent bilateral relationships shaped among the parent organisations of each powertrain system for the period 2008–2016. The related raw data lists all the organisations which collaborated in the field of individual powertrain systems for the development of joint patents granted between 2008 and 2016.

An Excel file is included in the Supplementary appendix of this article, which contains all the raw data related to the collaborating organisations at individual level over 1985–1996, 1997–2007, and 2008–2016 as well as the entire period 1985–2016.

### 1.2 Data at mutual level

At mutual level, we explored the common network features for each pair of the powertrain systems, i.e. ICEV-BEV, HEV-BEV, and ICEV-HEV. After extracting and exploring through a total of 4702 common patents, the three common networks were found with a total of 102 unique parent organisations connecting vis-à-vis 384 bilateral relationships and operating around 303 joint patents.

Table 3 shows the absolute and relative number of the joint patents shared between two powertrain systems over the entire period. An Excel file is included in the Supplementary appendix of this article, which contains the absolute and relative number of joint patents shared at mutual level between 1985 and 2016.

**Table 3:** The absolute and relative number of shared joint patents at mutual level (1985–2016).

Year	ICEV-BEV	HEV-BEV	ICEV-HEV	ICEV-BEV%	HEV-BEV%	ICEV-HEV%
1985	0	0	0	0.00%	0.00%	0.00%
1986	0	0	0	0.00%	0.00%	0.00%
1987	0	0	0	0.00%	0.00%	0.00%
1988	0	0	0	0.00%	0.00%	0.00%
1989	0	0	0	0.00%	0.00%	0.00%
1990	0	0	0	0.00%	0.00%	0.00%
1991	0	0	0	0.00%	0.00%	0.00%
1992	0	0	0	0.00%	0.00%	0.00%
1993	0	0	0	0.00%	0.00%	0.00%
1994	0	0	0	0.00%	0.00%	0.00%
1995	1	0	1	50.00%	0.00%	50.00%
1996	0	0	0	0.00%	0.00%	0.00%
1997	1	1	2	25.00%	25.00%	50.00%
1998	1	1	1	33.33%	33.33%	33.33%
1999	0	2	1	0.00%	66.67%	33.33%
2000	0	3	5	0.00%	37.50%	62.50%
2001	0	0	2	0.00%	0.00%	100.00%
2002	0	0	8	0.00%	0.00%	100.00%
2003	0	0	11	0.00%	0.00%	100.00%
2004	0	1	18	0.00%	5.26%	94.74%
2005	0	1	14	0.00%	6.67%	93.33%
2006	0	1	21	0.00%	4.55%	95.45%
2007	1	6	24	3.23%	19.35%	77.42%
2008	0	2	21	0.00%	8.70%	91.30%
2009	0	1	15	0.00%	6.25%	93.75%
2010	5	6	27	13.16%	15.79%	71.05%
2011	1	5	14	5.00%	25.00%	70.00%
2012	3	10	15	10.71%	35.71%	53.57%
2013	0	4	4	0.00%	50.00%	50.00%
2014	2	5	6	15.38%	38.46%	46.15%
2015	0	2	4	0.00%	33.33%	66.67%
2016	0	18	5	0.00%	78.26%	21.74%

Sum	15	69	219	4.95%	22.77%	72.28%

Table 4 shows the absolute and relative number of the bilateral relationships shared between two powertrain systems over the entire period. An Excel file is included in the Supplementary appendix of this article, which contains the absolute and relative number of bilateral relationships shared at mutual level between 1985 and 2016.

**Table 4:** The absolute and relative number of shared bilateral relationships at mutual level (1985–2016).

Year	ICEV-BEV	HEV-BEV	ICEV-HEV	ICEV-BEV%	HEV-BEV%	ICEV-HEV%
1985	0	0	0	0.00%	0.00%	0.00%
1986	0	0	0	0.00%	0.00%	0.00%
1987	0	0	0	0.00%	0.00%	0.00%
1988	0	0	0	0.00%	0.00%	0.00%
1989	0	0	0	0.00%	0.00%	0.00%
1990	0	0	0	0.00%	0.00%	0.00%
1991	0	0	0	0.00%	0.00%	0.00%
1992	0	0	0	0.00%	0.00%	0.00%
1993	0	0	0	0.00%	0.00%	0.00%
1994	0	0	0	0.00%	0.00%	0.00%
1995	1	0	1	50.00%	0.00%	50.00%
1996	0	0	0	0.00%	0.00%	0.00%
1997	1	1	2	25.00%	25.00%	50.00%
1998	6	1	1	75.00%	12.50%	12.50%
1999	0	0	1	0.00%	0.00%	100.00%
2000	0	2	5	0.00%	28.57%	71.43%
2001	0	0	2	0.00%	0.00%	100.00%
2002	0	0	10	0.00%	0.00%	100.00%
2003	0	0	13	0.00%	0.00%	100.00%
2004	0	1	18	0.00%	5.26%	94.74%
2005	0	1	19	0.00%	5.00%	95.00%
2006	0	0	23	0.00%	0.00%	100.00%
2007	3	6	26	8.57%	17.14%	74.29%
2008	0	2	35	0.00%	5.41%	94.59%
2009	0	3	15	0.00%	16.67%	83.33%
2010	19	6	31	33.93%	10.71%	55.36%
2011	6	5	14	24.00%	20.00%	56.00%
2012	8	10	17	22.86%	28.57%	48.57%
2013	0	4	4	0.00%	50.00%	50.00%
2014	21	5	6	65.63%	15.63%	18.75%
2015	0	2	4	0.00%	33.33%	66.67%
2016	0	18	5	0.00%	78.26%	21.74%

Sum	65	67	252	16.93%	17.45%	65.63%

**Fig. 4:**
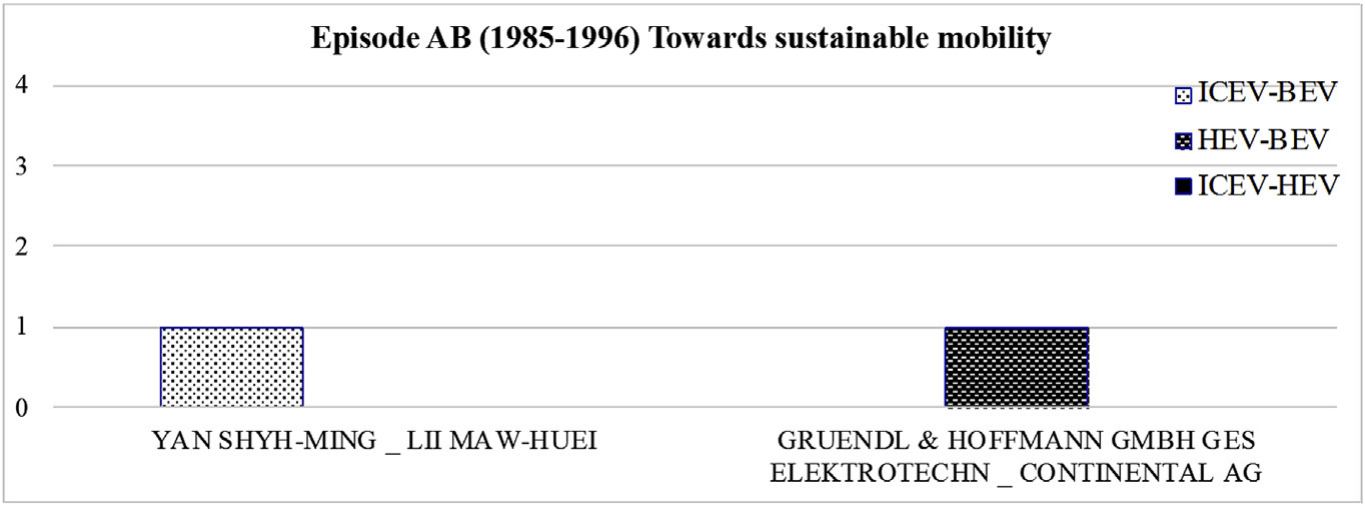
The most frequent shared bilateral relationships at mutual level for the period 1985–1996.

Fig. 4 displays the most frequent bilateral relationships shaped among the parent organisations of two powertrain systems for the period 1985–1996. The related raw data lists all the organisations that were in collaboration for the development of joint patents shared between two powertrain systems over 1985–1996.

**Fig. 5:**
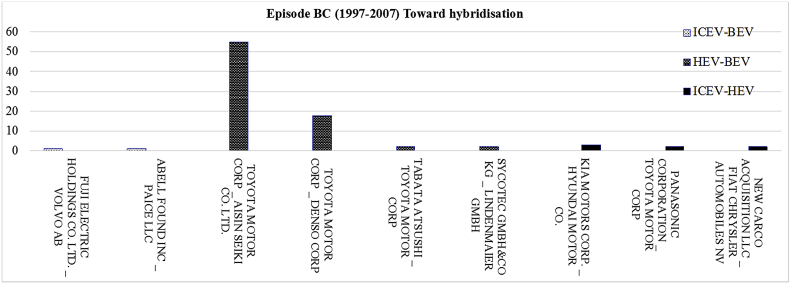
The most frequent shared bilateral relationships at mutual level for the period 1997–2007.

Fig. 5 displays the most frequent bilateral relationships shaped among the parent organisations of two powertrain systems for the period 1997–2007. The related raw data lists all the collaborating organisations which developed the joint patents shared between two powertrain systems over 1997–2007.

**Fig. 6:**
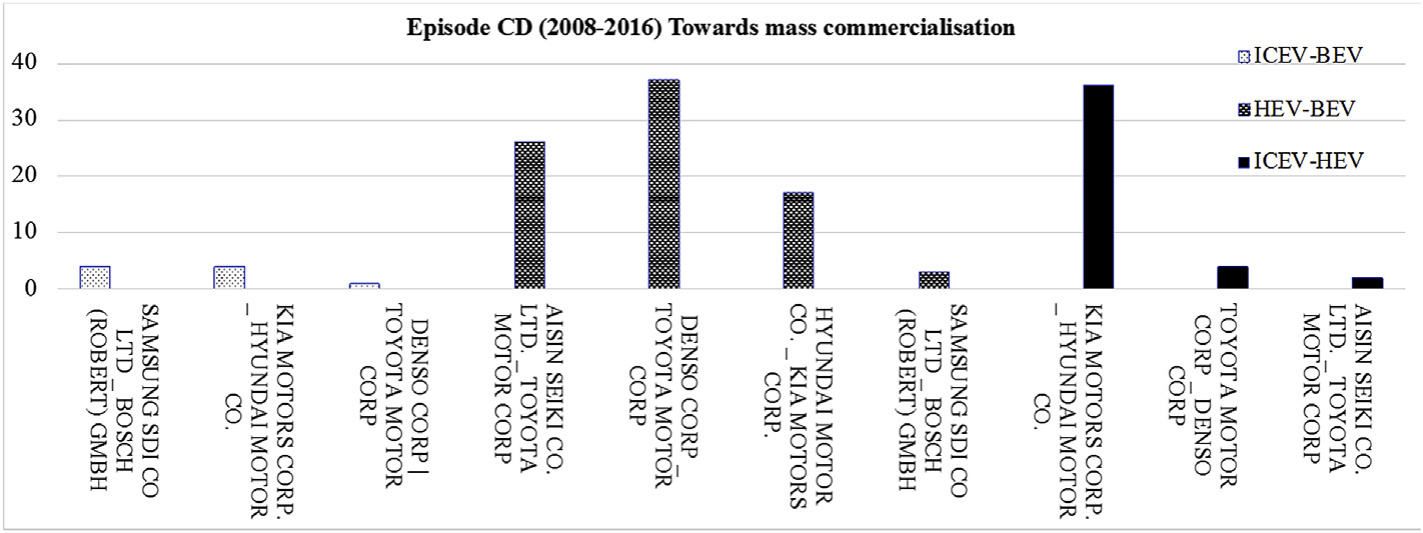
The most frequent shared bilateral relationships at mutual level for the period 2008–2016.

Fig. 6 displays the most frequent bilateral relationships shaped among the parent organisations of two powertrain systems for the period 2008–2016. The related raw data lists all the organisations that collaboratively developed the joint patents shared between two powertrain systems over 2008–2016.

An Excel file is included in the Supplementary appendix of this article, which contains all the raw data related to the collaborating organisations at mutual level over 1985–1996, 1997–2007, and 2008–2016 as well as the entire period 1985–2016.

Regarding environmental results, we discovered that the shared bilateral relationships between the powertrain systems were developed in total around 435 unique subgroup-level IPC codes, of which 134 subgroup-level codes were related to environmentally friendly innovations.

Table 5 shows whether a subgroup-level IPC code at mutual level is green for the period 1985–1996. Table 6 shows whether a subgroup-level IPC code at mutual level is green (or environmentally friendly) for the period 1997–2007. Table 7 shows whether a subgroup-level IPC code at mutual level is green for the period 2008–2016. The raw data related to Table 5, Table 6, Table 7 contain the frequency and the environmental nature of all the subgroup-level IPC codes that have been used in the joint patents shared between two powertrain systems over 1985–1996, 1997–2007, and 2008–2016, respectively. An Excel file is included in the Supplementary appendix of this article, which lists the frequency and the environmental nature of all the subgroup-level IPC codes used at mutual level over 1985–1996, 1997–2007, and 2008–2016 as well as the entire period 1985–2016.

**Table 5:** The frequency and the environmental nature of the subgroup-level IPC codes at mutual level for the period 1985–1996.

ICEV-HEV			HEV-BEV			BEV-ICEV		
IPCs	green	freq.	IPCs	green	freq.	IPCs	green	freq.

B60K0006485	yes	1	B60K000620	yes	3	B60K000626	yes	1
B60L000728	yes	1	B60K000626	yes	3	B60W001008	yes	1
B60L001114	yes	1	B60L001112	yes	3			
B60W001008	yes	1	B60L001114	yes	3			
B60W002000	yes	1	B60K000636	yes	2			
B60K001722	no	1	B60K000640	yes	2			
F02B007506	no	1	B60K0006448	yes	1			
F02N001104	no	1	F02B006100	no	1			
F16F001518	no	1	H02K0007116	no	1			
G05D001902	no	1	H02K000718	no	1			
H02K004902	no	1						
H02P001500	no	1						
H02P002900	no	1						

	Total: 13 Green: 5 Non-green: 8			Total: 20 Green: 17 Non-green: 3			Total: 2 Green: 2 Non-green: zero

**Table 6:** The frequency and the environmental nature of the subgroup-level IPC codes at mutual level for the period 1997–2007.

ICEV-HEV			HEV-BEV			BEV-ICEV		
IPCs	green	freq.	IPCs	green	freq.	IPCs	green	freq.

B60W002000	yes	62	B60W002000	yes	10	B60K000102	yes	2
B60W001006	no	57	B60K000620	yes	8	B60K000620	yes	2
B60K0006445	yes	55	B60W001006	no	8	B60K000646	yes	2
B60W001008	yes	52	B60W001008	yes	7	B60L001112	yes	2
B60L001114	yes	50	B60W001026	yes	7	B60L001118	yes	2
F02D002902	no	44	F02D002902	no	7	B60W001008	yes	2
B60W001010	no	41	B60L001118	yes	6	B60W002000	yes	2
B60K0006547	yes	38	B60L001114	yes	5	B60W001026	no	2
B60K000652	yes	30	B60K000646	yes	4	B60K000626	yes	1
B60K0006448	yes	29	B60K000648	yes	4	B60K000628	yes	1
B60W001004	no	27	F02D002906	no	4	B60K000632	yes	1
F16H006168	no	23	B60L001102	yes	3	B60K000636	yes	1
F16H0061684	no	22	B60L001112	yes	3	B60K0006365	yes	1
B60W001026	yes	21	H01M001044	yes	3	B60K0006442	yes	1
F16H0061686	no	21	H02J000700	yes	3	B60K000648	yes	1
F16H006350	no	21	H02J000714	yes	3	B60K0006485	yes	1
B60W001011	no	19	B60K000102	yes	2	B60K0006547	yes	1
B60K001704	no	17	B60K000626	yes	2	B60L001114	yes	1
B60K000654	yes	16	B60K000628	yes	2	B60L001520	yes	1
F02D002900	no	15	B60K000640	yes	2	F01N000320	yes	1
B60W0010115	no	14	B60K0006442	yes	2	F02D004100	yes	1
F16H000372	yes	12	B60K0006485	yes	2	H02J000700	yes	1
B60K0006365	yes	11	B60K0006543	yes	2	H02J000714	yes	1
B60K000648	yes	10	B60K0006547	yes	2	H02J000734	yes	1
F02D004500	yes	10	B60R001604	yes	2	B60H000100	no	1
B60W001000	no	10	B60K001704	no	2	B60H000132	no	1
F16H005914	no	10	B60L000300	no	2	B60T000832	no	1
F16H006102	no	10	B60W001002	no	2	B60W001006	no	1
F16H006340	no	10	B60W001004	no	2	B60W001018	no	1
B60K000102	yes	9	B60W001010	no	2	B60W001028	no	1
F16H006104	no	9	B60W001018	no	2	F02B003700	no	1
B60W001018	no	8	B60W001030	no	2	F02B003716	no	1
…	…	…	…	…	…			

	Total: 1061 Green: 521 Non-green: 540			Total: 150 Green: 94 Non-green: 56			Total: 47 Green: 32 Non-green: 15

**Table 7:** The frequency and the environmental nature of the subgroup-level IPC codes at mutual level for the period 2008–2016.

ICEV-HEV			HEV-BEV			BEV-ICEV		
IPCs	green	freq.	IPCs	green	freq.	IPCs	green	freq.

B60W002000	yes	68	B60W001008	yes	21	B60L001118	yes	6
B60W001006	no	55	B60W002000	yes	20	B60W002000	yes	5
B60K0006445	yes	52	B60W001006	no	18	B60W001006	no	4
B60W001008	yes	46	B60L001118	yes	11	B60W001008	yes	3
B60L001114	yes	45	B60L001114	yes	8	H02J000714	yes	3
F02D002902	no	40	B60W002015	yes	8	B60L000900	no	3
F02D004500	yes	23	B60K000648	yes	7	B60W003018	no	3
B60K0006547	yes	20	B60W003020	no	7	G06F000700	no	3
B60W001010	no	16	F02N001108	no	6	B60L001100	yes	2
B60K000648	yes	15	B60L001520	yes	5	B60R0016033	yes	2
B60K000652	yes	15	B60W001002	no	5	H02J000700	yes	2
B60W001026	yes	13	B60W003018	no	5	B60W001026	no	2
B60K0006448	yes	12	B60L000714	yes	4	G06F001700	no	2
B60W001004	no	11	B60L000718	yes	4	B60K000624	yes	1
F02D002906	no	10	B60L001112	yes	4	B60K0006445	yes	1
B60K0006365	yes	9	H02J000714	yes	4	B60K000648	yes	1
F16H006102	no	9	F02D002902	no	4	B60K0006547	yes	1
F16H006350	no	9	B60K0006445	yes	3	B60L001112	yes	1
B60K000640	yes	8	B60W001026	yes	3	B60L001114	yes	1
B60K000654	yes	8	B60W002013	yes	3	H01M000202	yes	1
F02D004114	yes	8	H02J000700	yes	3	H01M000210	yes	1
B60W001030	no	8	B60L000300	no	3	H01M000234	yes	1
B60W001002	no	7	B60L000900	no	3	H01M00100525	yes	1
F02N001108	no	7	B60W001010	no	3	H01M001046	yes	1
B60K000102	yes	6	B60W001030	no	3	H02J000704	yes	1
B60L001118	yes	6	F02N001104	no	3	B60L000100	no	1
F16H000372	yes	6	F16H005704	no	3	B60L000102	no	1
B60K001704	no	6	B60K000620	yes	2	B60W001002	no	1
B60L000300	no	6	B60K000626	yes	2	B60W001011	no	1
B60W003018	no	6	B60K0006387	yes	2	B60W0010115	no	1
F02N001104	no	6	B60L000710	yes	2	B60W003019	no	1
F16H0061686	no	6	B60L001100	yes	2	B60W005014	no	1
…	…	…	…	…	…	…	…	…

	Total: 937 Green: 501 Non-green: 436			Total: 300 Green: 168 Non-green: 132			Total: 70 Green: 52 Non-green: 28	

Regarding IPCs overlaps, Table 8 shows the frequency with which two powertrain systems share a group-level IPC code at mutual level for the period 1985–1996. Table 9 shows the frequency with which two powertrain systems share a group-level IPC code at mutual level for the period 1997–2007. Table 10 shows the frequency with which two powertrain systems share a group-level IPC code at mutual level for the period 2008–2016. The raw data related to Table 8, Table 9, Table 10 contain the frequency of all the group-level IPC codes that have been used in the joint patents shared between two powertrain systems over 1985–1996, 1997–2007, and 2008–2016, respectively. An Excel file is included in the Supplementary appendix of this article, which lists the frequency of all the group-level IPC codes used at mutual level over 1985–1996, 1997–2007, and 2008–2016 as well as the entire period 1985–2016.

**Table 8:** The most frequently shared used group-level IPC codes at mutual level for the period 1985–1996.

ICEV-HEV	freq.	HEV-BEV	freq.	BEV-ICEV	freq.
B60K0006	1	B60K0006	11	B60K0006	1
B60L0007	1	B60L0011	6	B60W0010	1
B60L0011	1	H02K0007	2		
B60W0010	1	F02B0061	1		
B60W0020	1				
F02B0075	1				
F02N0011	1				
F16F0015	1				
G05D0019	1				
H02K0049	1				
H02P0015	1				
H02P0029	1				
B60K0017	1				
	Total:13		Total:20		Total:2

**Table 9:** The most frequently shared used group-level IPC codes at mutual level for the period 1997–2007.

ICEV-HEV	freq.	HEV-BEV	freq.	BEV-ICEV	freq.
B60W0010	274	B60W0010	35	B60K0006	13
B60K0006	247	B60K0006	33	B60W0010	7
F16H0061	97	B60L0011	18	B60L0011	5
F02D0029	66	F02D0029	14	H02J0007	3
B60W0020	62	B60W0020	10	F02B0037	3
B60L0011	56	H02J0007	7	H01G0011	2
F16H0059	41	H01M0010	5	B60W0020	2
F16H0063	32	F02B0037	3	B60H0001	2
B60K0017	21	B60H0001	2	B60K0001	2
F16H0003	21	B60K0001	2	B60T0008	1
F02D0041	15	B60K0017	2	H02J0001	1
F02N0011	13	B60L0003	2	H02M0003	1
B60K0001	11	B60R0016	2	B60L0015	1
F02D0045	10	G01C0021	2	F01N0003	1
F16H0048	7	B60K0025	1	F02D0041	1
B60L0015	7	B60L0015	1	F02M0035	1
H02K0007	5	B60T0008	1	F02N0011	1
B60T0007	4	B60W0030	1		
B60T0008	4	F01N0003	1		
F02D0017	4	F02D0041	1		
F16H0057	4	F02D0045	1		
F02B0037	4	F02M0035	1		
F02M0025	4	F02N0011	1		
B60L0003	3	H01G0011	1		
B60L0009	2	H02J0001	1		
F02B0039	2	H02K0007	1		
F16H0045	2	H02P0009	1		
B60H0001	2				
H02K0005	2				
…	…				
	Total:1061		Total:150		Total:47

**Table 10:** The most frequently shared used group-level IPC codes at mutual level for the period 2008–2016.

ICEV-HEV	freq.	HEV-BEV	freq.	BEV-ICEV	freq.
B60K0006	190	B60W0010	62	B60W0010	12
B60W0010	169	B60W0020	39	B60L0011	10
B60W0020	73	B60L0011	27	H01M0010	8
B60L0011	57	B60K0006	23	H02J0007	6
F02D0029	56	B60W0030	17	B60W0020	5
F02D0041	37	B60L0007	12	B60W0030	4
F16H0061	36	F02N0011	12	B60K0006	4
F02D0045	23	H01M0008	10	H01M0002	3
F02N0011	15	H01M0010	10	B60L0009	3
F16H0059	15	B60L0015	7	G06F0007	3
F16H0063	14	H02J0007	7	C25D0011	3
B60W0030	12	B60L0003	5	G06F0017	2
F16H0003	11	F02D0029	5	B60L0001	2
B60K0017	9	B60W0050	4	B60R0016	2
B60K0001	8	F16H0057	4	F02N0011	2
B23K0026	7	H01M0004	4	H02M0001	1
B60L0003	7	B60L0001	3	H01F0037	1
B60L0009	6	B60L0009	3	H02J0001	1
H02J0007	6	F02D0041	3	B60W0050	1
B60W0050	6	F16H0061	3	H02P0009	1
F01N0003	6	H01M0002	3	H01F0038	1
B60L0015	5	B60K0017	2	F16H0057	1
G06F0019	4	B60N0002	2	H01F0027	1
F02D0017	4	B60R0016	2	G05D0003	1
F02M0025	4	B60T0008	2	F16H0061	1
G06F0017	4	F02B0039	2	G05D0001	1
F02B0053	4	G06F0007	2		
F16H0057	4	H02P0009	2		
F02M0026	4	B60K0026	1		
…	…	…	…		
	Total:937		Total:300		Total:80

## 2 Experimental design, materials, and methods

We collected the patent data from Thomson Reuters' online web-based platform Derwent Innovations Index [2], which is known as one of the largest and most prestigious patent platforms compiling data from over 80 global granting authorities [1]. Data collection occurred in November of 2018. We segmented the data into the three major automotive periods of ‘towards sustainable mobility’ (1985–1996), ‘towards hybridisation’ (1997–2007), and ‘towards mass commercialisation’ (2008–2016) [1]. We performed separate methodological steps for collecting and processing data at the individual and mutual levels.

### 2.1 Methodological steps at individual level

At individual level, we first extracted the patents related to each powertrain technological field from the DII platform using a combined search strategy of keywords IPC codes and keywords [1,3], shown in Table 11. Such strategy avoided any patents unrelated to the field [[4], [5], [6]]. We processed the data based on ‘patent families’ in order to avoid the multiple counting of the same inventions in different national patenting systems in the world [4,6]. We, additionally, ordered the extracted patents based on the earliest priority date in patent families. Because the priority date is the closest date to the finishing time of an invention that has been submitted for the first time to any of the world’ patenting systems [7], which can avoid including any additional lags, normally 18 months on average [5,7].

**Table 11:** Search terms of keywords and IPC codes used at individual level [1,3].

	Technological field	Search query
Individual level	ICEV-related patents	TAB=((“internal combustion engine” OR “ic engine” OR “diesel engine”) AND (vehicle* or car or automobile*)) AND (PRDS>=(19850101) AND PRDS<=(20161231)) AND IC=(F01* OR B60* OR F02B* OR F02D* OR F02F* OR F02 M* OR F02 N* OR F02P*);
	HEV-related patents	TAB=(“hybrid electric vehicle” OR “hybrid vehicle” OR “hybrid propulsion” OR “hybrid car” OR “hybrid automobile” OR “hybrid electric car”) AND (PRDS>=(19850101) AND PRDS<=(20161230)) AND IC=(F02* OR F16H* OR B60K006* OR B60W020 OR B60L00071* OR B60L000720)
	BEV-related patents	TAB=((“electric vehicle” OR “electric car” OR “electric automobile”) AND battery AND (vehicle* or car or automobile*)) AND (PRDS>=(19850101) AND PRDS<=(20161230)) AND IC=(H02k* OR H01 M* OR B60L011* OR B60L003* OR B60L015* OR B60K00101* OR B60W001008 OR B60W001024 OR B60W001026)

In the second step, we verified the quality and appropriateness of our patents data by running a manual validity check for at least 5% of our total patents [4]. We considered a patent valid for our database if its claim could contain “… the categorized technology as well as the possibility of an automotive utilization” [4, p79]. Table 12 shows that our manual validity check at individual level reached a good performance as the quality result for each powertrain system was above 85.00%. In the last step, we selected the patents that were jointly shared between two or more organisations or assignees, i.e. joint patents. Note that we counted only those organisations that were shown by the Thomson Reuters platform as ultimate parents. A joint patent shows whether the property rights of the invention are jointly assigned or owned by two or more organisations [8]. We took into account all the possible relationships in a joint patent by splitting any trilateral, quadrilateral or higher connections into bilateral relationships [1,9]. For instance, a patent co-assigned by Mitsubishi, Toyota, and Denso contains the three bilateral connections of Toyota- Mitsubishi, Toyota-Denso, and Mitsubishi-Denso.

**Table 12:** Validity check of data at individual level (1985–2016) [1].

		Granted patents		Validity check	
		Absolute	Relative	Sample size	Quality
Individual level	ICEV	49,154	62.43%	2460	87.25%
	HEV	10,888	13.83%	545	89.80%
	BEV	18,690	23.74%	940	88.25%
	Total	78,732	100.00%	3945	87.84%

### 2.2 Methodological steps at mutual level

At mutual level, we first extracted the patents shared between two powertrain technological fields from the DII platform using a different combined search strategy of IPC codes and keywords [1,3], shown in Table 13. We similarly processed the data based on ‘patent families’ and ‘priority date’.

**Table 13:** Search terms of keywords and IPC codes used at mutual level [1,3].

Technological field	Search query
ICEV-HEV related patents	TAB=((“internal combustion engine” OR “ic engine” OR “diesel engine”) AND (“hybrid electric vehicle” OR “hybrid vehicle” OR “hybrid propulsion” OR “hybrid car” OR “hybrid automobile” OR “hybrid electric car”) AND (vehicle* or car or automobile*)) AND (PRDS>=(19850101) AND PRDS<=(20161231)) AND IC=(F01* OR B60* OR F16H × OR F02B* OR F02D* OR F02F* OR F02 M* OR F02 N* OR F02P* OR B60K006* OR B60W020 OR B60L00071* OR B60L000720)
ICEV-BEV related patents	TAB=((“internal combustion engine” OR “ic engine” OR “diesel engine”) AND (“electric vehicle” OR “electric car” OR “electric automobile”) AND (vehicle* or car or automobile*)) AND (PRDS>=(19850101) AND PRDS<=(20161230)) AND IC=(F01* OR B60* OR F02B* OR F02D* OR F02F* OR F02 M* OR F02 N* OR F02P* OR H02k* OR H01 M* OR B60L011* OR B60L003* OR B60L015* OR B60K00101* OR B60W001008 OR B60W001024 OR B60W001026)
BEV-HEV related patents	TAB=((“electric vehicle” OR “electric car” OR “electric automobile”) AND battery AND (vehicle* or car or automobile*) AND (“hybrid electric vehicle” OR “hybrid vehicle” OR “hybrid propulsion” OR “hybrid car” OR “hybrid automobile” OR “hybrid electric car")) AND (PRDS>=(19850101) AND PRDS<=(20161230)) AND IC=(F16H* OR H02k* OR H01 M* OR B60L011* OR B60L003* OR B60L015* OR B60K00101* OR B60W001008 OR B60W001024 OR B60W001026 OR B60K006* OR B60W020 OR B60L00071* OR B60L000720)

In the second step, we verified their quality and appropriateness by another manual validity check for 5% of the total shared patents. As Table 14 shows, the quality at mutual level reached a good performance as well. In the third step, while we selected only those patents that were jointly assigned to two or more organisations (i.e. shared joint patents), we again split any trilateral, quadrilateral or higher connections in a joint patent into bilateral relationships (i.e. shared bilateral relationships). In the fourth step, we explored the overlaps between the powertrain systems in terms of environmental innovations and knowledge domains by extracting the IPC codes that were used within the shared joint patents. We used IPC codes for two reasons. First, IPC codes are able to manifest the knowledge domains overlaps between patents because the IPC codes (knowledge domains) used in a patent do not exist solely for the development of the intended invention but can be exploited and utilised for other inventions [10]. Second, IPC codes are able to manifest whether innovations built in an invention are environmentally friendly (or green) [1,11]. Note that for the environmental innovations overlap we took advantage of subgroup-level IPC codes (e.g. B60W-010/10) as they can distinguish green innovations from non-green ones. The IPC green inventory adopted by Ref. [11] was used, which is a combination of the WIPO's IPC Green Inventory and the OECD's list of environmentally-sound technologies (EST). For the knowledge domain overlap, we reduced the extracted subgroup-level IPC codes to group-level IPC codes (e.g. B60W-010) as they can provide more general but useful information about the technical or knowledge domains of an invention [1,12].

**Table 14:** Validity check of data at mutual level (1985–2016).

	Granted patents		Validity check	
	Absolute	Relative	Sample size	Quality
ICEV-HEV	3486	74.14%	175	86.29%
HEV-BEV	849	18.06%	43	88.37%
BEV-ICEV	367	7.81%	19	89.47%
Total	4702	100.00%	237	86.50%
